# Fibroblasts selectively modulate immune cell-derived cytokine effects on tumor cells in colorectal cancer

**DOI:** 10.3389/fimmu.2026.1891700

**Published:** 2026-07-29

**Authors:** Szabolcs Hajdó, Zsolt I. Komlósi, Barbara Érsek, Idan Carmi, Tamás Tölgyes, Zoltán Wiener

**Affiliations:** 1 Institute of Genetics, Cell- and Immunobiology, Semmelweis University, Budapest, Hungary; 2 Department of General Surgery and Surgical Oncology, Uzsoki Hospital, Budapest, Hungary

**Keywords:** CAF, IFNγ, IL-17, IL-22, innate lymphoid cells, MAPK, NK cells, patient-derived organoid

## Abstract

**Introduction:**

Colorectal cancer (CRC) is a heterogeneous malignancy and a major cause of cancer-related mortality worldwide. Cancer-associated fibroblasts (CAFs) accumulate in tumors and correlate with poor patient survival, suggesting a central role in immune regulation. Patient-derived organoids (PDO) maintain the intra-tumoral cellular heterogeneity of the original tissue, thus, they represent one of the best methods to study human cancers. The tumor microenvironment (TME) contains diverse immune cell populations, including innate lymphoid cells (ILCs), yet how stromal components influence cytokine-driven tumor–immune interactions remains unclear.

**Methods:**

PDOs from CRC patients were used to screen TME-derived cytokines affecting tumor growth. Organoid-forming efficiency and signaling pathway activation were analyzed following cytokine stimulation in the presence or absence of CAFs. Tumor-infiltrating ILC subsets were characterized in patient samples, and co-culture systems were employed to assess cytokine production and stromal–immune interactions.

**Results:**

IL-22 was identified as a cytokine that increased PDO-forming efficiency without activating fibroblasts. Although IL-22 typically signals through the JAK–STAT pathway, it unexpectedly activates MAPK signaling in PDO cells. Interestingly, interferon-γ (IFNγ) showed only partial cytotoxic effects on CRC cells. Tumor tissues contained both IFNγ-producing ILC1 and IL-22/IL-17A–producing ILC3 populations. Co-culture with PDOs selectively induced IL-22, but not IL-17A, production in ILC3 cells. Importantly, IL-22 enhanced organoid formation only in the absence of CAFs, whereas IFNγ activity was largely unaffected by stromal context.

**Discussion:**

These findings demonstrate that CAFs modulate local immunity by selectively masking ILC3-derived IL-22 signaling while preserving ILC1-mediated IFNγ responses. This study emphasizes the importance of stromal context in interpreting cytokine function in CRC and reveals a previously unrecognized mechanism of stromal–immune crosstalk within the TME.

## 1 Introduction

Colorectal cancer (CRC) is a heterogeneous disease and one of the leading causes of cancer-related death. While the microsatellite unstable (MSI) subtype has significant cytotoxic T cell infiltration, microsatellite stable (MSS) CRC cases display a locally immunosuppressive environment in the tumor tissue that is critically shaped by stromal cells (1). The transcriptomics-based Consensus Molecular Subtype (CMS) classification of CRC identified an aggressive subgroup (CMS4) characterized by the accumulation of cancer-associated fibroblasts (CAFs), epithelial-mesenchymal transition (EMT), and lacking effective curative treatment (2, 3). Previous studies have shown that high CAF levels correlate with poor patient survival (4).

Recently, a tumor–stroma biobank from CRC patients has been established. Analysis of this biobank revealed that fibroblasts play a critical role in influencing the sensitivity of CRC organoids to several therapies, including the chemotherapeutic drug 5-fluorouracil (5-FU) and epidermal growth factor receptor (EGFR) inhibitors (5). These effects are mostly mediated through modulation of the local tumor microenvironment (TME), also called the niche, which is central to CRC progression. The niche includes extracellular matrix (ECM) components, mainly produced by CAFs, as well as immune cells, cytokines, and growth factors. Among the immune cells of TME some lymphocyte subpopulations have been identified relatively recently: the innate lymphoid cells (ILCs), which are enriched in mucosal tissues like the gut (6). ILCs are classified into ILC1, ILC2, and ILC3 subsets based on their transcription factor expression, cytokine production after cytokine-induced stimulation. While ILC1 and ILC2 cells are major producers of IFNγ and IL-4, respectively, ILC3 cells mainly produce IL-17A and IL-22. ILCs play an important role in maintaining immune homeostasis at barrier sites, contributing to antimicrobial immunity and epithelial regeneration. However, their impact on tumor development is less well understood, largely due to the dynamic nature of ILC subsets and the complex cytokine responses within the TME (7, 8). Notably, contradictory data on how the ratio of ILC1 and ILC3 cells changes in CRC compared to the normal colon, and to the extent to which these cells contribute to tumorigenesis have been reported (9, 10). Despite the recognized importance of immune cells within and surrounding tumor tissue, the mechanisms by which fibroblast accumulation modulates the effects of immune cell-derived cytokines remain poorly understood.

In this study, we employed organoid technology to examine how niche factors influence CRC in the presence and absence of CAFs, with a focus on their role in modulating immune responses. Specifically, we investigated the contributions of ILC-derived IL-22 and IFNγ, both of which play key roles in patient-derived organoid (PDO) formation. Although literature data showed that IL-22 typically signals through the JAK–STAT pathway, it unexpectedly promotes tumor organoid formation via MAPK signaling, and this effect is observed only in the absence of CAFs. In contrast, IFNγ exhibits a partial direct anti-tumor effect that is independent of fibroblast presence. Overall, CAFs exert a dominant influence by promoting tumor growth and selectively overriding IL-22-mediated effects, highlighting their central role in shaping the CRC microenvironment.

## 2 Materials and methods

### 2.1 Human CRC organoid cultures and quantification of organoid number and size

We used CRC organoid lines isolated and published by our research group (see the organoid characterization and patient data in (11, 12) and **Supplementary Table 1**). Organoids were cultured in “full CRC medium” composed of advanced DMEM/F12 (Thermo Fisher Scientific, Waltham, MA, USA), 10 mM HEPES (Merck, Darmstadt, Germany), glutamine, B27 supplement (1:50, Thermo Fisher Scientific), primocin (1:500, Invivogen, San Diego, CA, USA), 10 mM nicotinamide (Merck), 1 mM N-acetyl-cysteine (Merck), and 40 ng/mL EGF (Peprotech). To prevent anoikis, the Rho kinase inhibitor Y27632 (10 µM, Merck) was added for 3 days after passaging the organoids. In some experiments, EGF and Y27632 were omitted from the medium (noEGF medium). For co-cultures with NK/ILC cells, a simple CRC medium was used that contained no EGF, Y27632, nicotinamide and N-acetyl-cysteine. During passaging, organoids were mechanically removed from the 3D matrix (Geltrex, Thermo Fisher Scientific) every 5–7 days, they were centrifuged at 850×g for 7 minutes, washed with PBS, and digested with TrypLE (Thermo Fisher) until dissociated into small cell clusters. When testing the organoid forming efficiency, PDOs were dissociated into single cell suspension. In some experiments, cells were treated with the MEK inhibitor trametinib (MedChemExpress, 1µM), human EGF, IFNγ (Peprotech), IL-22 or IL-1α (Immunotools, Friesoythe, Germany). For low-scale screening, HGF, IL-11, IL-17A, IL-22, IL-1β (50 ng/mL, Immunotools), IFNγ (50 ng/mL, Peprotech), TGFβ (10 ng/mL, Peprotech), TNFα (50 ng/mL, Peprotech), WNT3A or WNT5A (100 ng/mL, Peprotech) were applied. Unless otherwise indicated, experiments with IL-22 were carried out without EGF.

CRC organoids were imaged at 10× magnification on an inverted phase contrast microscope (Nikon), and their largest diameters were measured using the ImageJ software (National Institutes of Health, USA). For each experimental group, 15–20 organoids were analyzed. Organoid numbers were counted in phase contrast microscope.

### 2.2 Cell cultures

Normal human colon fibroblasts (CCD-18Co, CRL-1459, ATCC, Manassas, VA, USA), SW1222 (RRID: CVCL_3886), HT29 (RRID: CVCL_0320), HCT116 (RRID: CVCL_0291) cell lines and CAFs (from patients #1, 2, 4, 6, see also (13)) were cultured in DMEM with 4500 g/L glucose, GlutaMAX (Thermo Fisher Scientific), 10% FBS (Biosera, Kansas, MO, USA), 1% penicillin/streptomycin (Thermo Fisher), and 1% glutamine (Merck). We only used cells with low passage numbers (< p15).

### 2.3 Co-cultures

CCD-18Co colon fibroblasts or CAFs were dissociated into single cell solution. CRC organoids and fibroblasts or CAFs (10,000:20,000 cells in 20µL Geltrex, 1:2 ratio) were then mixed before embedding into Geltrex in noEGF medium. The following cocultures were established with CAFs: PDO#1-CAF#4, PDO#2-CAF#1, PDO#3-CAF#6, PDO#4-CAF#2. In some experiments, fibroblasts were incubated with Vybrant™ Cell-Labeling Solution DiD dye for 10 min following the Cell in Suspension protocol of Thermo Fisher, the cells were extensively washed with DMEM twice to remove the free dye, and they were further cultured for 24 h before co-culturing experiments. For organoid-NK cell co-cultures, PDOs were plated on Geltrex-coated wells, and isolated NK cells were added in simple CRC medium at approximately a 1:1 cell ratio. In the organoid and ILC3 co-cultures, PDOs were plated in Geltrex droplets, and isolated ILC3s were added in simple CRC medium at a fixed number (1000 ILC3 cells/well) with human IL-15 (1 ng/ml), IL-7 (20 ng/ml), and IL-2 (20 ng/ml, all from Immunotools) to support ILC survival.

### 2.4 Flow cytometry and cell sorting

Organoids were dissociated into single cells with TrypLE before flow cytometric measurements. When analyzing tumor tissue, samples were dissociated using the Tumor Dissociation Kit (Miltenyi Biotech, Bergisch Gladbach, Germany) with the gentleMACS Octo Dissociator following the manufacturer’s protocol (see clinical data of patients in **Supplementary Table 1**). For labelling, cells were suspended in FACS buffer (PBS + 1 mM EDTA, 25 mM HEPES, 1% bovine serum albumin (BSA) and incubated with antibodies for 20 minutes. For detecting intracellular antigens, cells were fixed in 4% paraformaldehyde (PFA) in PBS for 20 minutes. They were then permeabilized with 0.1% saponin, and then labeled with antibodies. 5,000–10,000 events were measured using a Cytoflex (Beckman Coulter, Brea, CA, USA) instrument. For ILC cell sorting, we used the Sony SH800S cell sorter after labelling (Sony Biotechnology, Bothell, WA, USA). For NK cell isolation, an NK cell isolation kit (130-092-657, Miltenyi Biotech, Bergisch Gladbach, Germany) was used. When analyzing peripheral blood of healthy donors (age-matched to the cancer patients), we obtained buffy coats from the National Blood Service of Hungary, we isolated PBMCs with Histopaque-1077 (Merck) and density gradient centrifugation as a source of ILCs and NK cells. The antibodies used are listed in **Supplementary Table 2**. In some experiments, cells dissociated from organoids were incubated with the LIVE/DEAD™ Fixable Near-IR Dead Cell Stain Kit (Thermo Fisher) for 30 minutes following the manufacturer’s protocol and then analyzed by Cytoflex.

### 2.5 RNA isolation and gene expression analysis

We isolated total RNA using the miRNEasy Micro Kit (Qiagen) according to the manufacturer’s description in 15 µL of RNAse-free water, we measured the RNA purity and concentration with a NanoDrop instrument (Thermo Fisher), and we then prepared cDNA from 300–500 ng of RNA (in 20 µL final volume) using the Sensi-FAST cDNA Synthesis Kit (Bioline, Meridian Bioscience, London, UK). Quantitative PCR was carried out with the SensiFAST Sybr No-Rox Kit (Bioline) using a BioRad CFX384 Touch real-time PCR instrument (Hercules, CA, USA, 384-well format, 5 µL per well) with the SybrGreen method at an annealing temperature of 60 °C. Results were calculated using the equation: relative expression level = 2^-ΔCt^, where ΔCt = Ct(gene of interest) - Ct(housekeeping gene). The primers are listed in **Supplementary Table 3**.

### 2.6 ELISA

Conditioned medium from confluent normal colon fibroblast, CAF cultures (in serum-free medium), ILC3 cells cultured in 3D matrix or ILC3 and PDO co-cultures was collected after 48 hours. Cell debris was removed by centrifugation at 300g for 5 min, and IL-22 measurements were performed according to the manufacturer’s protocol (Human IL-22 Uncoated ELISA Kit, 88-7522-88, Thermo Fisher). We measured the OD values on a HiPo MPP-96 Microplate Photometer (Biosan, Riga, Latvia).

### 2.7 Whole-mount immunostaining

Normal colon fibroblasts were cultured in 8-well chamber slides (BD Biosciences, East Rutherford, NJ, USA), fixed in 4% PFA for 30 min, washed with PBS, and blocked and permeabilized with blocking buffer (5% FBS, 0.2% BSA, 0.3% Triton X-100 in PBS) for 20 minutes. Samples were incubated with primary antibodies at 4 °C overnight, with secondary antibodies for 2 hours at room temperature in blocking buffer, and then mounted with ProLong Diamond antifade mountant containing DAPI (Thermo Fisher Scientific). Confocal images were taken with a Leica TCS SP8 microscope, and ImageJ software was used for image analysis. We examined 5–6 images per microscopic field using a 20x objective. Antibodies are listed in **Supplementary Table 2**.

### 2.8 Bioinformatical analysis

The GSE17537 and GSE14333 data sets were unified and z-score transformed before carrying out survival analysis by Kaplan–Meier plotting and log-rank test. nCPM (Normalized Counts Per Million) data for T-cells and innate lymphoid cells were extracted from the single cell RNA-seq data of Protein Atlas (proteinatlas.org) for IL-22. For further single cell RNA-seq analysis, we loaded MMSp samples from the GSE178341 dataset (https://www.ncbi.nlm.nih.gov/gds/?term=GSE178341, containing mismatch repair proficient CRC samples) into the Icarus_v3 analysis software (The University of Auckland). Pre-filtering and pre-analysis steps were carried out with default settings. For dimensionality reduction, we used the lognormalize method with 50 dimensions, and for clustering, we set the number of dimensions and UMAP parameters to 20. Cell labels were assigned with the “Sctype reference cell marker data” and with “immune system” as tissue type.

### 2.9 Statistical analysis

Student’s paired or unpaired t-tests were applied with *p < 0.05, **p < 0.01 and ***p < 0.005 significance levels and with Microsoft Excel and GraphPad softwares. When not otherwise indicated, original data were used for statistical evaluation. Mean + SD values are shown with replicates indicated with dots. When quantifying whole-mount immunostaining, 5–6 images were analyzed for each condition.

## 3 Results

### 3.1 IL-22 activates the MAP kinase pathway in CRC cells

**Figure 1:**
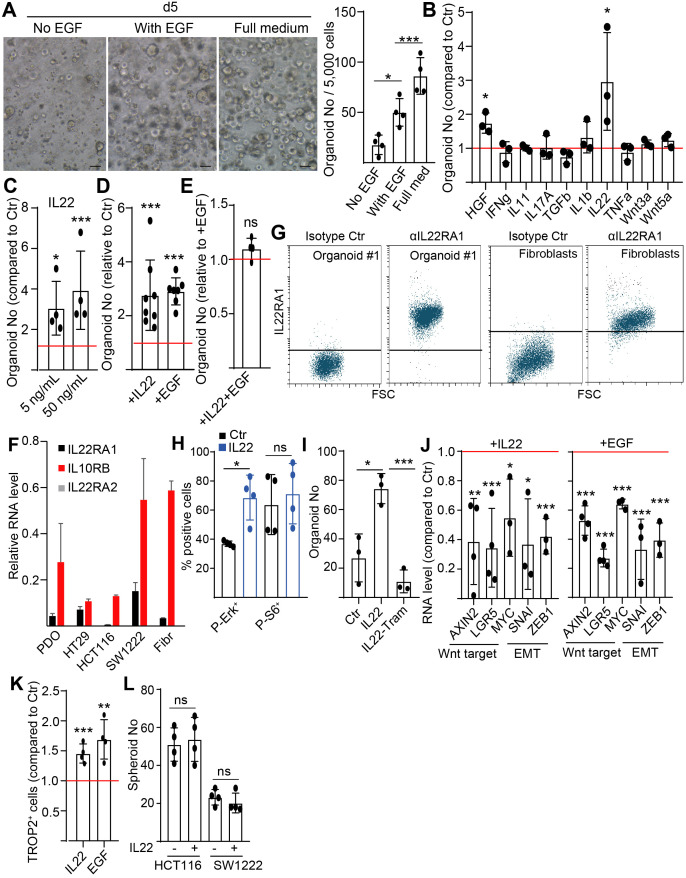
IL-22 promotes PDO proliferation through the MAPK pathway. **(A)** Representative light microscopy images of PDO cultures in EGF-free (No EGF), EGF-supplemented (With EGF) and full medium (containing both EGF and Rho-kinase inhibitor) and their quantification (day 5). **(B)** Comparison of relative organoid numbers across the indicated treatments (applied in medium lacking EGF) compared to untreated controls shown with a red line), determined on day5. **(C, D)**. Relative organoid numbers with different IL-22 concentrations **(C)** and different treatments (EGF, IL-22: 50 ng/mL, day5) **(D)** compared to untreated controls. **(E)** Comparison of organoid numbers with IL-22+EGF treatment versus EGF alone. **(F)** Expression levels (normalized to housekeeping gene) of the indicated genes in PDOs, HT29, HCT116, SW1222 cell lines, and normal human colon fibroblasts (RT-qPCR, n=4 for PDOs, n=4 CRC cell lines and n=3 for fibroblasts). **(G)** Representative flow cytometry images showing IL-22RA1 expression in one PDO line and normal colon fibroblasts. The horizontal line was set according to the isotype controls. **(H)** Percentage of p-Erk^+^ and p-S6^+^ cells within PDOs in the presence and absence of IL-22 (day 3, flow cytometry, with no EGF). **(I)** Number of organoids with the indicated treatments (day 5, IL-22: 50 ng/ml, trametinib (Tram): 1µM). **(J)** Relative expression levels of Wnt target genes (AXIN2, LGR5, MYC) and EMT marker genes (SNAI, ZEB1) in PDOs treated with IL-22 or EGF for 24h (RT-qPCR). RNA levels were normalized to housekeeping and then compared to the untreated controls (red line). **(K)** Relative percentage of TROP2^+^ cells in PDOs with IL-22 or EGF treatments (analysis of flow cytometry results, day 3). **(L)** Number of spheroids formed in HCT116 and SW1222 cell lines with or without IL-22 treatment (day 5). Note that these cell lines do not depend on EGF. Each dot represents one PDO line in the figure. In D, PDO lines were measured twice. Scale bars: 50µm. Unpaired t-test **(A, H, I, L)** or paired t-test **(B–E, J, K)** were used with ***p<0.005, **p<0.01, *p<0.05 and ns, p>0.05.

To assess the impact of niche-derived factors, we performed a small-scale screen in patient-derived organoids (PDOs) using molecules previously implicated in CRC tumorigenesis. Since activation of the MAP kinase pathway is critical in tumorigenesis and organoid formation, and we focused on exogenous factors of stromal origin, we used PDO lines that were dependent on exogenous EGF signaling (14). As expected, EGF and the Rho-kinase inhibitor that prevents anoikis, were the most efficient combination inducing organoid formation, and EGF alone also resulted in a marked increase in the organoid number (**Figure 1A**). We next examined the effects of selected factors on CRC organoid formation in reduced medium lacking EGF, starting from single-cell suspensions. Due to the low efficiency of organoid formation in the absence of EGF, this test was suitable to detect molecules that increase the number of novel organoids. In line with this notion, IFNγ, a known major effector cytokine in various tumors, had no significant effect on organoid number in EGF-free medium (**Figure 1B**). While several niche factors had no detectable inducing effect on organoid formation, both hepatocyte growth factor (HGF) and IL-22 significantly increased organoid-forming efficiency (**Figure 1B**). Interestingly, IL-22 acted already at a low concentration, it mimicked the effect of EGF (**Figures 1C, D**), and its effect on organoid-forming efficiency completely disappeared in the presence of EGF (**Figure 1E**). Notably, single cell RNA-seq data suggested that IL-22 is produced by T-cells and innate lymphoid cells at various levels in different tissues (**Supplementary Figure 1A**), and we observed IL-22 in a cluster of immune cells containing lymphocytes in CRC-derived datasets, too (**Supplementary Figure 1B**).

Since HGF is produced by CAFs and is a well-established activator of the MAPK pathway, we first focused in our subsequent analyses on another molecule, IL-22. Expression analyses revealed the presence of both IL-22 receptor subunits, IL22RA1 and IL10RB, not only in PDOs but also in the CRC cell lines SW1222, HT29 and HCT116, as well as in fibroblasts (**Figure 1F**). In contrast, IL22RA2, which encodes a soluble decoy receptor that inhibits IL-22 signaling, was expressed at only marginal levels relative to IL22RA1 across all cell types examined (**Figure 1F**). Importantly, flow cytometric analysis confirmed cell surface expression of IL22RA1 on both PDO cells and fibroblasts (**Figure 1G**). Together, these findings indicate that IL-22 can act directly on PDOs, CRC cell lines, and fibroblasts.

To further study the effect of IL-22, we applied this cytokine in cultures with already established organoids. Notably, we observed no significant difference in organoid diameter, percentage of proliferating or dying cells (**Supplementary Figure 1C**). Additionally, IL-22 exposure did not alter the proportion of EpCAM^+^ epithelial cells within the organoids (**Supplementary Figure 1D**).

Phosphorylation of ribosomal protein S6 (p-S6) and phospho-ERK1/2 (p-ERK) serve as readouts of mTORC1 and MAPK pathway activation, respectively. Interestingly, while IL-22 did not affect the percentage of p-S6^+^ cells, it significantly increased the fraction of p-ERK^+^ cells when EGF was lacking (**Figure 1H**). Furthermore, treatment with the MEK inhibitor trametinib, which blocks MAPK signaling, completely diminished CRC organoid formation even in the presence of IL-22 (**Figure 1I**), indicating that IL-22 does not induce MAPK-independent escape mechanisms. Consistently, IL-22 elicited gene expression changes that were highly similar to those induced by EGF in PDOs (**Figure 1J**). Similarly to EGF, IL-22 increased the percentage of TROP2^+^ cells that is a marker of the high-plasticity tumor cell state in CRC (15) (**Figure 1K**). Collectively, these data indicate that IL-22 promotes CRC organoid initiation by activating the MAPK pathway. Supporting this conclusion, although IL-22 increased organoid-forming efficiency in EGF-dependent PDOs, it had no effect on spheroid formation in SW1222 and HCT116 cells which proliferate independently of EGF signaling (**Figure 1L**).

### 3.2 No major effect of IL-22 on the polarization and gene expression of fibroblasts

We previously identified a CD142^high^ CAF population with pronounced pro-tumorigenic activity (13). Independently, others have described an IL-1R^high^ CAF subset in CRC (16) induced by IL-1α and IL-1β, corresponding to inflammatory CAFs (iCAFs) in pancreatic ductal adenocarcinoma (PDAC) and CRC (17). In contrast, myofibroblastic CAFs (myCAFs) are characterized by αSMA expression, they represent a major source of ECM components, and are polarized by TGFβ across multiple tumor types (17). As expected, IL-1α treatment increased the proportion of IL-1R^high^ fibroblasts, while having no effect on the frequency of CD142^high^ cells (**Supplementary Figures 2A, B**). Moreover, co-culture of PDOs with fibroblasts reduced the relative abundance of iCAFs within the 3D matrix (**Supplementary Figures 2A, B**), consistent with previous findings that close proximity to tumor cells promotes fibroblast polarization from an iCAF toward a myCAF state (17).

Notably, IL-22 treatment did not alter the proportion of either CD142^high^ or IL-1R^high^ fibroblast populations (**Supplementary Figure 2C**). In addition, only marginal changes were observed in the expression of key CAF-derived secreted factors, including TGFβ1, IL-1β, IL-11, and HGF (**Supplementary Figure 2D**), all of which are known to play critical roles in shaping the tumor microenvironment. Consistent with these findings, IL-22 had no effect on the TGFβ-induced αSMA expression in fibroblasts (**Supplementary Figure 2E**), indicating a lack of myCAF polarization. Collectively, these data demonstrate that IL-22 does not alter the balance between iCAFs and myCAFs, nor between CD142^high^ and CD142^low^ fibroblast populations.

### 3.3 ILC3 cells express IL-22, but not IL-17A when co-cultured with tumor cells

**Figure 2:**
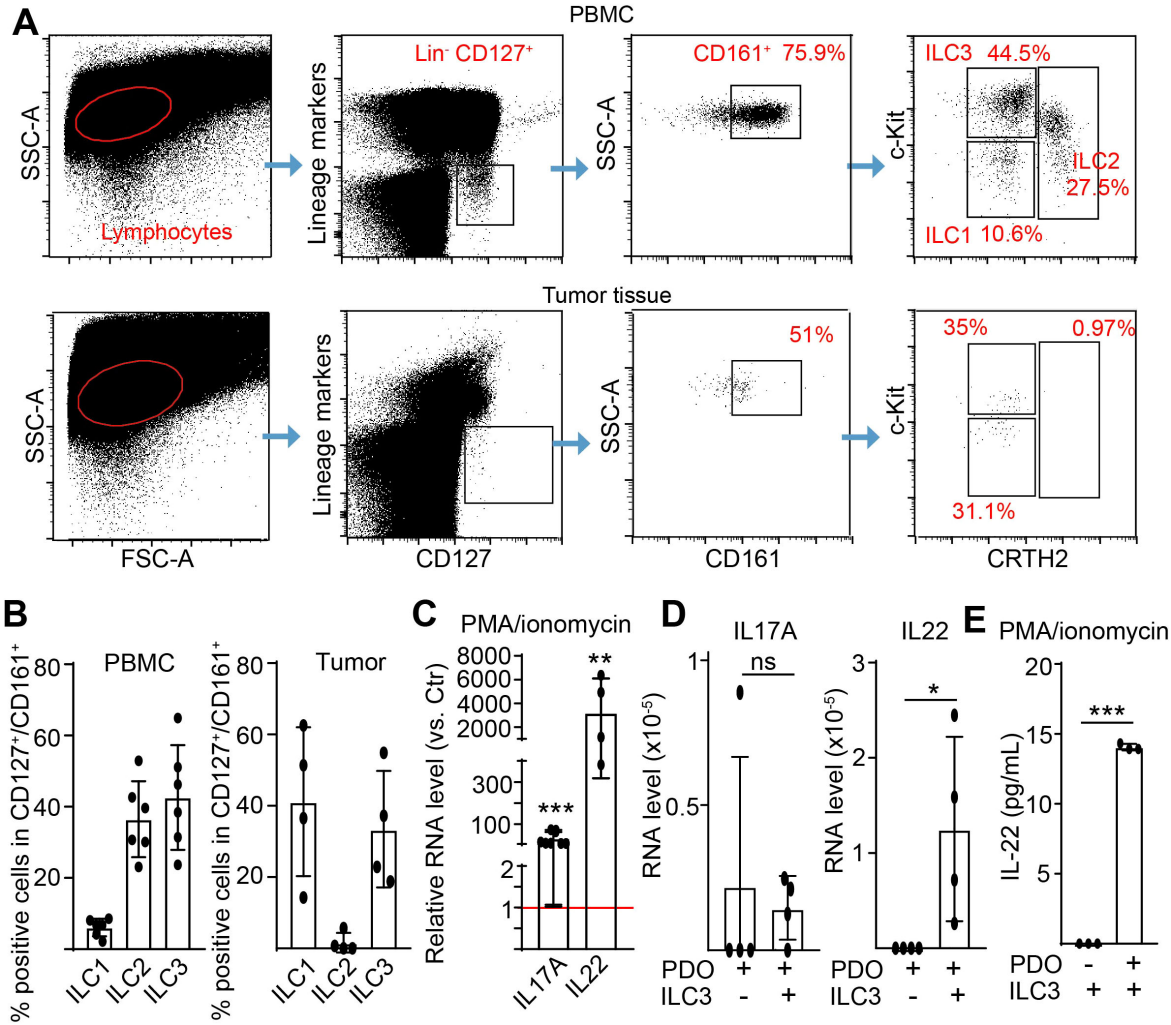
ILC3 cells are present in tumor tissue and produce IL-22 in co-cultures with PDOs. **(A)** Representative flow cytometry dot plots from PBMC and tumor tissue illustrating the gating strategy to identify ILCs. Surgical tumor samples (n=4) and PBMC (n=6) were measured. ILCs were characterized as Lineage^-^CD127^+^CD161^+^ cells within the lymphocyte gate (lineage markers: see **Supplementary Table 2**). ILC2 cells were defined as CRTH2^+^cKit^+/-^, ILC3 cells as CRTH2^-^cKit^+^, and ILC1 as CRTH2^-^cKit^-^. **(B)** The distribution of the three ILC subtypes across PBMC (healthy controls) and tumor samples (flow cytometry). **(C)** Relative expression levels of IL17A and IL22 in PMA (30 ng/mL)/ionomycin (1 µg/mL)-stimulated ILC3 cells (day 2, RT-qPCR). RNA levels were normalized to the housekeeping and then compared to the unstimulated controls (red line). **(D)** Expression of the indicated genes in single PDO and in PDO-ILC3 co-cultures (RT-qPCR). Note the lack of expression in PDO monocultures. RNA levels normalized to the housekeeping gene are shown. **(E)** IL-22 level in the conditioned medium of PDO-ILC3 co-cultures and ILC3 cultures (ELISA, cells were cultured for 48 h and stimulated with PMA (30 ng/ml) and ionomycin (1 ug/ml) for 2h before collecting samples). Note that the values for ILC3 cultures were below the lowest detection limit. Each dot represents one patient sample **(B)** or one co-culture with a PDO line **(C, D, E)**. Unpaired **(D)** or paired **(E)** t-test or one-sample t-test on log2-normalized relative data **(C)** were applied with ***p<0.005, **p<0.01, *p<0.05 and ns, p>0.05.

Although some studies reported IL-22 expression in fibroblasts (18), we were not able to detect IL-22 protein in either normal colon fibroblasts or CAFs by ELISA (**Supplementary Figure 2F**). We therefore next investigated innate lymphoid cell type 3 (ILC3) populations as a potential source of IL-22. Notably, whereas all ILC populations were detectable in the peripheral blood of healthy donors, we could detect only ILC1 and ILC3 cells in freshly isolated CRC tissues (**Figures 2A, B**). After sorting ILC3 cells and their nonspecific stimulation with PMA-ionomycin, ILC3 cells produced both IL-22 and IL-17A *in vitro* (**Figure 2C**). However, only IL-22 expression was induced in co-cultures with PDOs (**Figure 2D**). In addition, organoids enhanced the IL-22 protein level in co-cultures in stimulated ILC3 cells (**Figure 2E**). These findings identify ILC3 cells as a relevant source of IL-22 within the tumor microenvironment.

**Figure 3:**
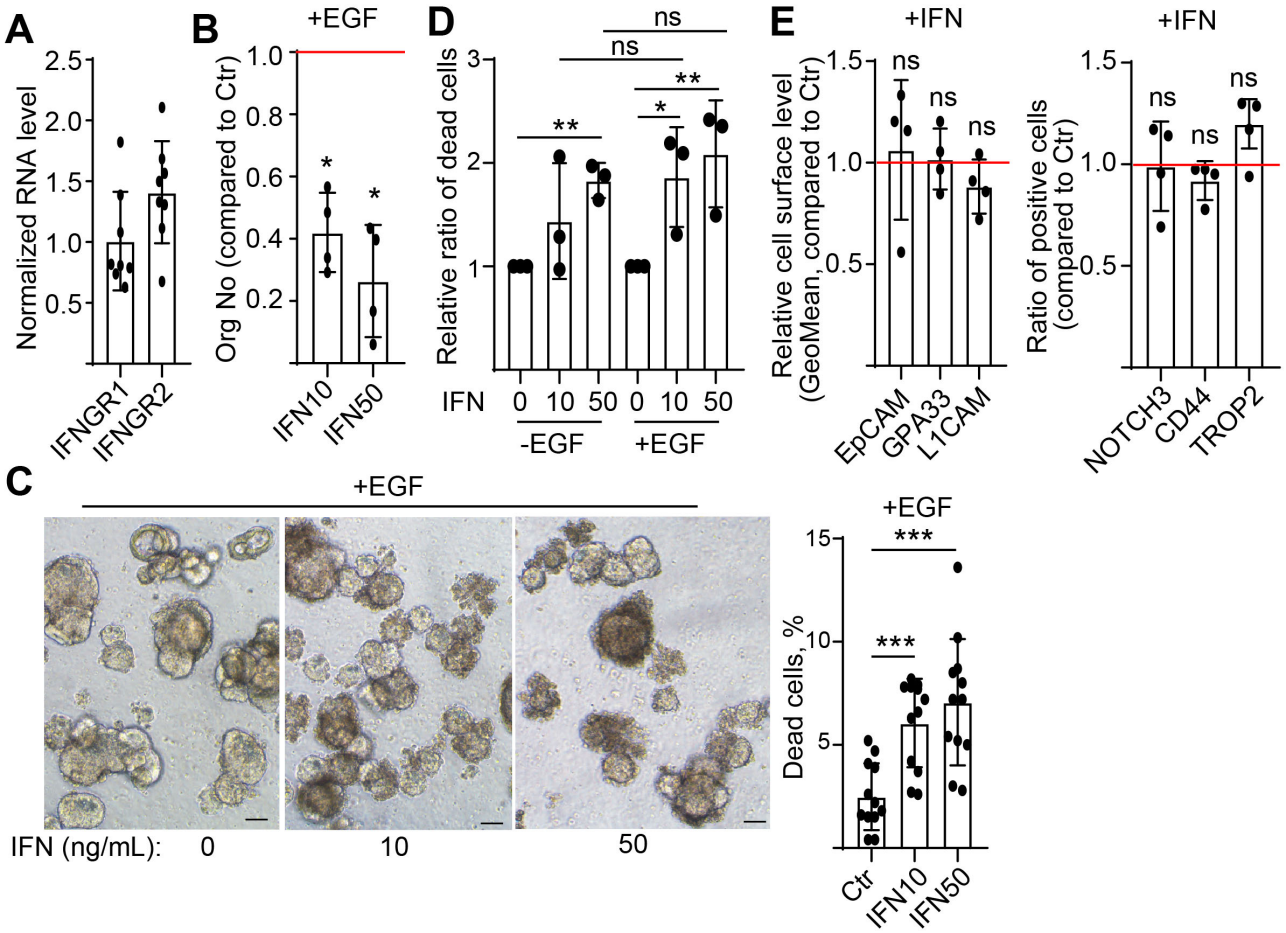
IFNγ triggers partial cell death in PDOs. **(A)** RNA levels of IGFR1 and IGFR2 in PDOs, normalized to housekeeping genes (RT-qPCR). **(B)** Relative organoid number (day 5) in IFNγ-treated cultures (10 and 50 ng/mL) compared to the absence of this cytokine (red line). Note the presence of EGF (50 ng/mL). **(C)** Representative light microscopy images of PDO cultures treated with increasing concentrations of IFNγ in the presence of EGF, and the quantification of dead cells with flow cytometry (day 4). Note the partially disintegrating organoids in the presence of IFNγ. **(D)** The ratio of dead cells within PDOs with or without EGF and in the absence or presence of IFNγ at different concentrations (flow cytometry). **(E)** Relative level of epithelial (EpCAM, GPA33, NOTCH3, CD44) and high cell plasticity (L1CAM, TROP2) markers under IFNγ treatment (flow cytometry). Expression levels are indicated by GeoMean fluorescence intensity (left) and the proportion of positive cells (right). Scale bars: 50µm **(C)**. Each dot represents one PDO line (A, B, D, E). In case of A and C, each PDO line was measured 2 or 3 times, respectively. Unpaired **(C, D)** or paired t-test **(B, D, E)** were used with *p<0.05, **p<0.01, ***p<0.005 and ns, p>0.05.

Interestingly, we observed an increased proportion of ILC1 cells in CRC tissue compared with peripheral blood (**Figure 2B**), whereas ILC2 cells were hardly detected in the tumors. To assess first the functional relevance of ILC1 enrichment, we examined the effects of IFNγ, the principal cytokine secreted by ILC1 cells. Both IFNGR1 and IFNGR2 were expressed in all PDO lines analyzed (**Figure 3A**), and IFNγ treatment had a modest, dose-dependent reducing effect on organoid-forming efficiency in the presence of EGF (**Figure 3B**). When applied to cultures containing already established organoids, IFNγ caused a modest and dose-dependent reduction in cell viability, however, many organoids at least partially survived (**Figure 3C**). Interestingly, this effect was observed both in the absence and presence of EGF (**Figure 3D**). Moreover, IFNγ exposure did not selectively affect specific tumor cell subpopulations, as evidenced by unchanged expression of epithelial markers (EpCAM, GPA33, CD44, NOTCH3), and markers of the high-plasticity cell state (TROP2, L1CAM) (**Figure 3E**). Taken together, whereas IL-22 derived from ILC3 cells may have a tumor supporting function, IFNγ produced by ILC1 cells partially reduces PDO cell survival.

### 3.4 IL-22 does not protect tumor cells against immune-mediated cytotoxicity

**Figure 4:**
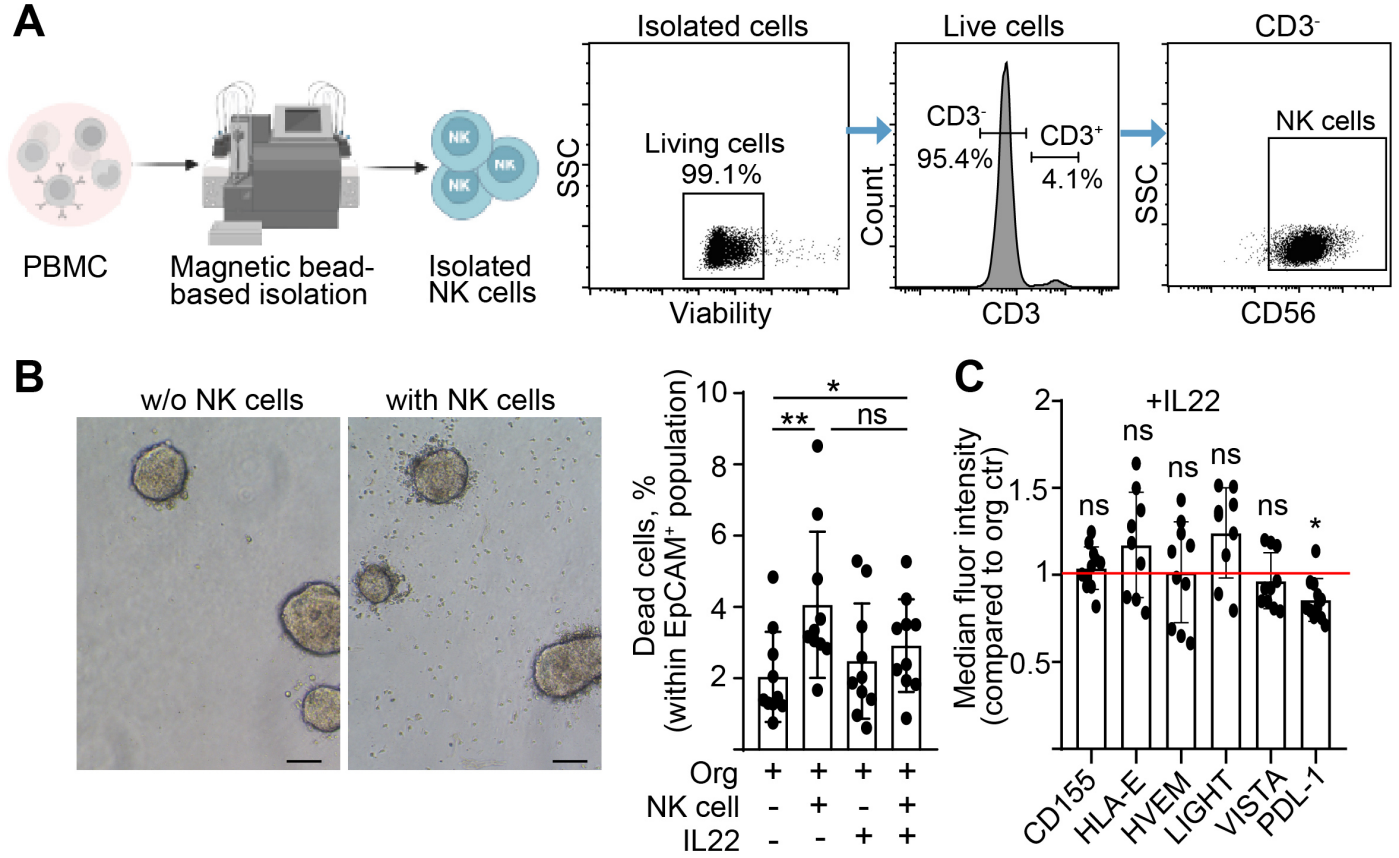
IL-22 does not significantly affect the immune evasion properties of PDOs. **(A)** Schematic illustration of the NK cell isolation process (left), and representative flow cytometry plots displaying the gating strategy to confirm isolation purity (right). Note that NK cells are CD3^-^CD56^+^. **(B)** Representative light microscopy images depicting organoids and co-cultures with NK cells (left), and the percentage of dead cells in monocultures or co-cultures with/without IL-22 (50 ng/mL for 3 days) detected with flow cytometry (right). Note that dead cells were detected only within the EpCAM^+^ tumor cells. **(C)** Signal intensity of specific NK cell receptor ligands in PDOs treated with IL-22 (day 3), compared to untreated controls (flow cytometry). Scale bars: 50µm **(B)**. Each dot represents one measurement, and each PDO line was studied 2–3 times **(B, C)**. Paired t-tests were used with **p<0.01, *p<0.05 and ns, p>0.05.

Several cytokines and metabolites are known to have tumor-promoting or immunosuppressive effects in the complex TME of solid tumors, leading to tumor growth or immune evasion, respectively. To test whether IL-22 can confer a protective effect against immune cell-mediated killing of PDOs, we isolated circulating NK cells, a major effector of cellular immunity, and co-cultured them with PDOs pre-treated with IL-22 (**Figure 4A**). We observed that NK cells exhibited only partial killing of PDOs (**Figure 4B**). Interestingly, IL-22 did not significantly increase the survival of CRC cells in the presence of NK cells compared to the untreated controls (**Figure 4B**). We also tested the expression of some major immune checkpoint molecules, NK cell receptor ligands, in IL-22-treated PDOs, including PD-L1, HLA-E, LIGHT, CD155, and VISTA, which could influence the effector function of cytotoxic immune cells, and found no major changes in their expression (**Figure 4C**).

### 3.5 Fibroblasts prevent the effect of IL-22, but not IFNγ on tumor cells

**Figure 5:**
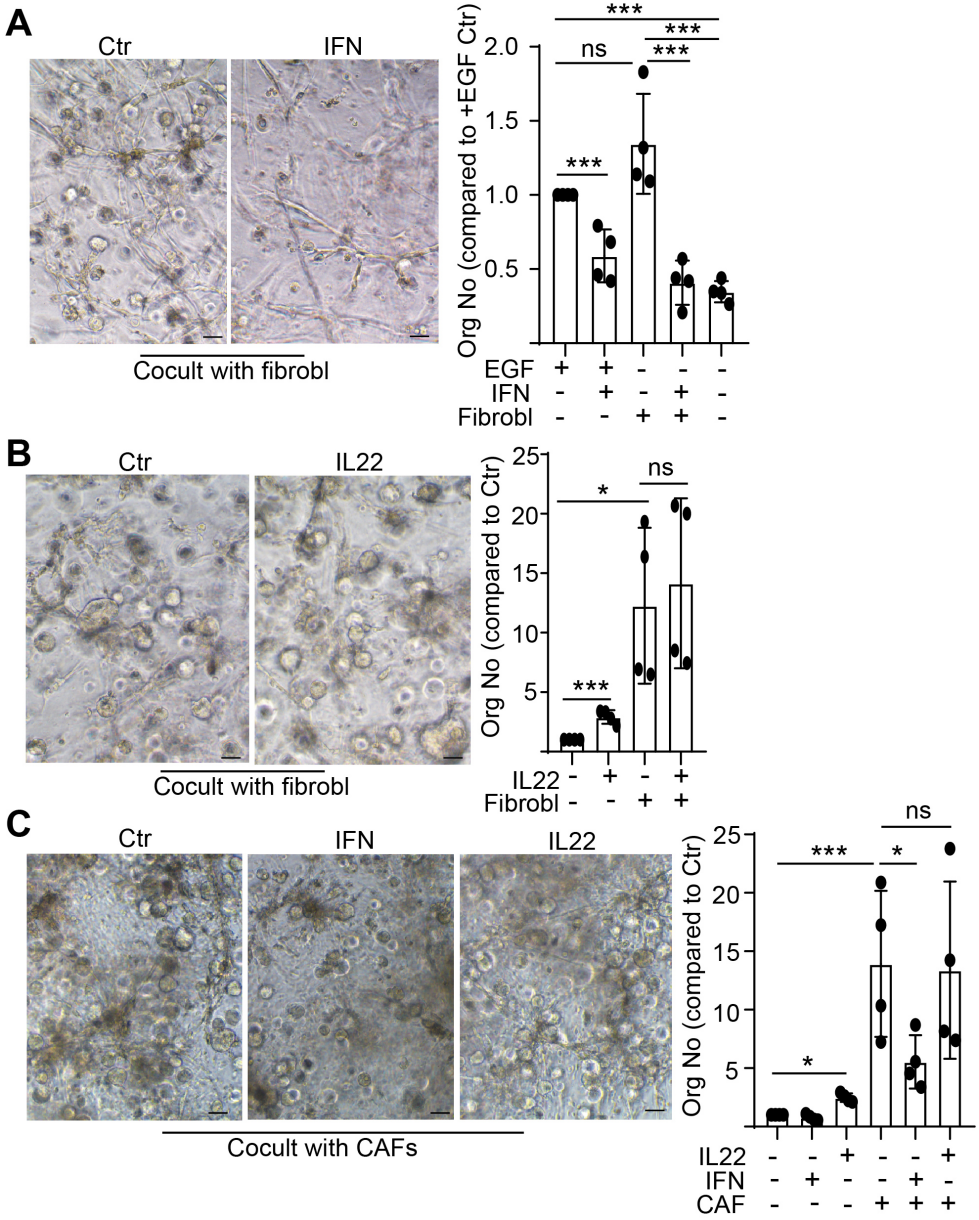
Fibroblasts override the effect of IL-22 but not IFNγ in the CRC tumor microenvironment. **(A)** Light microscopy images of organoid-fibroblast co-cultures with and without IFNγ (left). Organoid numbers were quantified with or without fibroblasts, EGF (50 ng/mL), and IFNγ (right panel, 10 ng/mL, 3 days). Note that IFNγ significantly reduced organoid numbers even when fibroblasts were present. **(B)** Light microscopy images showing organoid-fibroblast co-cultures with and without IL-22. Organoid numbers were measured with or without fibroblasts and/or IL-22 (50 ng/mL, right panel, in the absence of EGF). Note that in the presence of fibroblasts, IL-22 has no effect on the organoid number. **(C)** Co-cultures of CAF and PDO lines or only PDOs in the presence or absence of IL-22 or IFNγ (light microscopy images and their quantification, day 4). Scale bars: 50µm. Each dot represents one PDO line. Paired t-test was used with ***p<0.005, *p<0.05, ns, p>0.05.

To investigate how fibroblasts modulate the effects of immune cell-derived cytokines within the tumor microenvironment, we co-cultured them with PDOs. Since fibroblasts are a major source of EGF family members, organoid-forming efficiency in these co-cultures was similar to organoid mono-cultures with EGF (**Figure 5A**). While IFNγ reduced the organoid-forming efficiency (**Figure 5A**), the stimulatory effect of IL-22 on organoid formation was completely abrogated in the presence of fibroblasts (**Figure 5B**), and we confirmed these results in co-cultures of CAFs and PDOs, too (**Figure 5C**). Collectively, these findings indicate that MAPK pathway activity is essential for CRC organoid formation and that IL-22 promotes this process, at least in part, through MAPK signaling. While ILC-derived IFNγ and IL-22 exert opposing and relatively modest effects on tumorigenesis, slightly inhibitory and stimulatory, respectively, fibroblasts exert a critical influence that overrides the effect of IL-22 acting via the MAPK pathway. These observations provide a mechanistic explanation of how CAF accumulation modifies and suppresses the effect of the immune cell-derived cytokine microenvironment, which acts via MAPK in CRC. Consistent with this interpretation, expression levels of IL22 and IL22RA1 do not correlate with either good or poor patient survival (**Supplementary Figure 3A**). Furthermore, supporting the finding of only partial killing effect of IFNγ and its contradictory role in CRC, we observed a negative correlation between IFNGR2 and patient survival, but not for IFNγ or IFNGR1 (**Supplementary Figure 3B**).

## 4 Discussion

Colorectal cancer is characterized by pronounced molecular and cellular intra-tumoral heterogeneity. This heterogeneity is influenced by the tumor microenvironment containing altered ECM components, cytokines, innate and adaptive immune cells and CAFs. After carrying out a small-scale screen to identify critical niche factors of CRC tumorigenesis, we focused on IL-22 and IFNγ with the most profound effect on organoid-forming efficiency. Notably, although IL-22 is generally thought to act via the JAK-STAT pathway, we observed that it activates MAPK signaling in CRC cells. Although IL-22 receptor is also expressed on fibroblasts, we found no major effect of IL-22 on the polarization and gene expression of these cells. Furthermore, we proved that co-cultures of ILC3 and tumor cells induced the expression of IL22. We detected both ILC1 and ILC3 cells, major sources of IFNγ and IL-22, respectively, in CRC patient tumor samples. The accumulation of fibroblasts masked the colony-forming effect of IL-22, but they had no influence on the effects of IFNγ. Thus, these observations underscore the central role of fibroblasts in promoting tumor growth, but on the other hand, we also demonstrate that CAFs may not override the effects of important anti-tumoral effector cytokines, such as IFNγ.

The tumor microenvironment contains, among other cell types, cancer-associated fibroblasts (CAF) and immune cells, both secreting several cytokines and growth factors that may contribute to CRC maintenance and propagation. Out of the several factors tested, we demonstrated that under EGF-depleted conditions, only HGF, and notably IL-22, increased the colony-forming capacity of PDOs, which are inherently EGF dependent, whereas IFNγ reduced cell viability. While the pro-tumorigenic roles of EGF and HGF in CRC are well established, and these growth factors are primarily released by fibroblasts, the role of the immune cell-derived IL-22 has been mostly substantiated in colitis-associated cancer models so far (19–21). IL-22 receptor components are thought to be found on skin and mucosal barrier epithelial cells and fibroblasts, but are absent from immune cells (22). We confirmed the presence of IL-22 receptor in PDOs, CRC cell lines, and CAFs. Interestingly, IL-22 increased the organoid-forming efficiency, but did not significantly affect the size of already established organoids, and it only marginally influenced overall cell viability. Although we used a small number of PDO lines, these findings suggest that IL-22 primarily promotes the clonogenic capacity.

IL-22 is known to mainly act on the JAK-STAT signaling pathway, leading to the proliferation of epithelial cells, and in several colitis-associated CRC models, promoted tumorigenesis through STAT3 activation (22, 23). The importance of this pathway is illustrated by the fact that IL-22 and oncostatin M (OSM) form a pathogenic circuit that drives inflammation and tumorigenesis (19), and CRC tumor stem cells mediate anti-VEGF (bevacizumab) resistance through the IL-22-STAT3 signaling pathway (24). However, some studies demonstrated that IL-22 can also activate the MAPK and PI3K/AKT pathways (25). Although we did not study the JAK-STAT signaling in this work, we found that IL-22 stimulation induced ERK phosphorylation without increasing phosphorylation of S6, indicating the activation of the MAPK pathway but not mTOR signaling. Importantly, pharmacological inhibition of MEK with trametinib abrogated the IL-22-induced increase in colony formation, demonstrating that IL-22-driven organoid proliferation is MAPK-dependent. Notably, Ruan HX recently published the IL-22-induced ERK phosphorylation in HCT116 CRC cells (26). In contrast, we did not observe any effects of IL-22 in HCT116 or SW1222 cell lines (25, 26). Since these cell lines exhibit EGF-independent growth and display KRAS mutations (G13D in HCT116, and A146V in SW1222) leading to constitutive activation of downstream MAPK signaling, this is in line with our findings for the lack of effect of IL-22 on colony formation of these cell lines. Nevertheless, some of our data are supported by Ruan et al, showing the importance of the MAPK pathway in the effects of IL-22 (25). However, to our knowledge, we demonstrated for the first time that this finding is not restricted to CRC cell lines in 2D models only, but it is a more general mechanism that can be observed also in 3D organoids that represent the cellular heterogeneity of the original tumor tissue. Moreover, we showed the importance of IL-22-initiated MAPK activation in a more complex microenvironment, including stromal fibroblasts that are critical players in shaping tumor progression. In addition, the IL-22-induced MAPK activation is only important when other MAPK-activating factors, such as EGF family members, are lacking. Since CAFs are a major source of EGF family members and HGF, the accumulation of fibroblasts critically modifies the effect of the local cytokine microenvironment.

Several immune cell-derived cytokines are important in the chemoresistance of CRC cells. Of note, a large proportion of these data derive from *in vitro* studies with only 2D cell cultures that do not take into consideration the complex microenvironment and the presence of CAFs. CAFs are widely recognized for their pro-tumoral functions, including the regulation of tumor growth, invasion, and therapeutic resistance (27). Furthermore, the accumulation of CAFs is a major hallmark of the CRC subtype with the worst disease outcome (3). Molecular and phenotypic profiling have revealed that the tumor stroma of many solid tumors contains heterogeneous cancer-associated fibroblast (CAF) populations. Among these, two major subtypes, myofibroblastic CAFs (myCAFs) producing ECM proteins, and inflammatory CAFs (iCAFs) that are a source of inflammatory cytokines, were originally characterized in pancreatic ductal adenocarcinoma and are now recognized across multiple cancer types (16, 17, 28). IL-22 did not significantly alter the balance between iCAFs and myCAFs, nor did it affect the distribution of the tumor-promoting CD142^high^ fibroblast populations (13). Consistent with this, IL-22 had minimal impact on the gene expression of key CAF-derived mediators, indicating that it does not substantially reprogram fibroblast functional states. Collectively, these findings indicate that IL-22 exerts limited direct influence on CAF subset specification. Instead, its protumorigenic effects are more likely mediated through direct signaling in tumor cells rather than through modulation of fibroblast polarization.

IL-22 is secreted by both innate and adaptive immune cells, with ILC3s, Th17, and Th22 cells representing the principal sources. ILC3 cells secrete IL-17A and IL-22 upon IL-23 and IL-1β stimulation, contributing to intestinal epithelial homeostasis, tissue repair, and antimicrobial immunity (29). We detected ILC3s in CRC samples and demonstrated that, within our co-culture system with PDOs, they predominantly secreted IL-22 rather than IL-17A. ILC3s can be further subdivided into NCR^-^ and NCR^+^ cells based on their expression of natural cytotoxicity receptors (NCRs). NCR^-^ ILC3s produce both IL-17A and IL-22, whereas NCR^+^ ILC3s mainly secrete IL-22 (30, 31). Although we did not address these two subpopulations, our data may raise the possibility that CRC organoids preferentially activate an IL-22-secreting ILC3 subset. Notably, the relative distribution of ILC subtypes differed between the tumor tissues and peripheral blood, with only ILC1 and ILC3 cells being in the tumor microenvironment. Interestingly, other studies have reported enrichment of ILC1-like and ILC3 cells in CRC and other pathological conditions (32, 33). ILC1 cells, like Th1 cells, produce primarily IFNγ upon stimulation. Given that IFNγ can have either pro- or antitumorigenic effects depending on the cellular context (34, 35), this could explain our observation of its partial cytotoxic activity in the PDOs, and the negative correlation between IFNγ receptor expression and patient survival. To sum up, these findings suggest that ILC3 cells may represent relevant sources of IL-22 in the CRC microenvironment, whereas IFNγ does not exert a complete anti-tumor effect in our 3D *in vitro* model.

A previous study by Briukhovetska et al. reported immunosuppressive functions of IL-22 in a mouse model of lung and breast cancer, involving the induction of the immune checkpoint molecule CD155 in cancer cells, thereby reducing NK cell effector functions (36). However, in CRC, co-culture of NK cells with IL-22-conditioned PDOs revealed no protective effect of IL-22 and no significant alterations in the expression of key immune checkpoint molecules.

Although the aggressive (CMS4) subtype of CRC is particularly enriched in CAFs, transcriptomic analyses indicate that the presence of fibroblasts is a general feature across CRC subtypes (37, 38). Therefore, we intended to contextualize the effects of IL-22 and IFNγ in the CAF-enriched tumor stroma. When co-culturing PDOs with fibroblasts or CAFs, the pro-tumorigenic effect of IL-22 on organoid formation was no longer apparent, as fibroblasts alone markedly enhanced organoid proliferation. Importantly, CAFs are critical in producing several factors that induce the MAPK pathway in CRC cells, such as HGF or members of the EGF family (13). Interestingly, fibroblasts or CAFs could not substantially attenuate the anti-proliferative effects of IFNγ. Together, these findings indicate that fibroblasts or CAFs exert a dominant influence on CRC organoid behavior, effectively overriding IL-22-mediated effects, while having a limited impact on IFNγ-driven responses.

## 5 Conclusion

In summary, our data identify ILC3 cells as a potential source of IL-22 within the CRC microenvironment. Although according to previous literature data IL-22 generally acts via the JAK-STAT pathway, we also show that IL-22 promotes tumor organoid formation via activating the MAPK signaling, and only in the absence of CAFs. Conversely, IFNγ, produced partly by ILC1 cells, shows only limited direct anti-tumor activity, which is not modified by fibroblasts. Our data reveal that CAFs can overshadow the effects of cytokines using the MAPK pathway while sparing others, indicating that while both IL-22 and CAFs contribute to tumor cell proliferation, the influence of CAFs is dominant. Thus, these results demonstrate that CAFs not only stimulate tumor growth, but they selectively shape the immune microenvironment in CRC. Furthermore, the effects of individual cytokines on tumor behavior must be reinterpreted in the presence of stromal cells, such as fibroblasts.

## Data Availability

The GSE178341, GSE17537 and GSE14333 online available datasets were analyzed in this study.
