# Supplementary material for: Fibroblasts selectively modulate immune cell-derived cytokine effects on tumor cells in colorectal cancer
Source: Front Immunol. 2026 Jul 29;17:1891700. doi: 10.3389/fimmu.2026.1891700 (PMC13461434; doi:10.3389/fimmu.2026.1891700)
Supplement: Supplementary file 1 [file DataSheet1.pdf]

Fibroblasts selectively modulate immune cell–derived cytokine effects on tumor cells in colorectal cancer

Szabolcs Hajdó[1], Zsolt Komlósi[1], Barbara Érsek[1], Idan Carmi[1], Tamás Tölgyes[2], Zoltán Wiener[1]

A.

Sample	Gender	Age	Tumor	Differentiation grading	Stage	Nutlin sensitivity	EGF dependency
CRC1	F	63	colon adenocarcinoma	Grade 2	T3N0M0	no	yes
CRC2	M	74	colon adenocarcinoma	Grade 2	T3N0M0	no	yes
CRC3	M	75	colon adenocarcinoma	Grade 2	T3N2aM0	no	yes
CRC4	M	74	colon adenocarcinoma	Grade 2	T3N1M0	yes	yes

B.

Sample	Gender	Age	Tumor	Differentiation grading	Stage
CRC-CAF1	F	39	colon adenocarcinoma	Grade 2	T3N2bM1a
CRC-CAF2	F	37	colon adenocarcinoma	Grade 2	T3N1aM0
CRC-CAF4	F	56	colon adenocarcinoma	Grade 2	T3N1aM0
CRC-CAF6	M	68	colon adenocarcinoma	Grade 2	T3N0M0
CRC-CAF7	F	55	colon adenocarcinoma	Grade 2	T3N0M0
CRC-CAF8	M	71	colon adenocarcinoma	Grade 2	T3N1bM0

C.

Sample	Gender	Age	Tumor	Differentiation grading	Stage
Sample1	F	56	colon adenocarcinoma	Grade 1	pT3pN1a
Sample2	F	79	colon adenocarcinoma	Grade 1	pT4apN2a
Sample3	M	76	colon adenocarcinoma	Grade 1	pT4apN1a
Sample4	M	69	colon adenocarcinoma	Grade 1	pT4aN0

Table S1. Clinical data of our PDO lines (A), CAFs (B), and tumor samples used in our study (C). A. Note that all PDO lines are EGF-dependent (lack KRAS and BRAF mutations), and they contain TP53 mutations (sensitive to nutlin-3). For further detailed characterization of the organoid lines, see (1, 2) B. Clinical data of patients from whom CAFs were isolated (see also (3)). C. Patient data for flow cytometric measurement of ILCs in tumor samples

Antibody	**Producer**	**Cat No**	**FC/IC/cell sorting**
human CD34 Antibody FITC	*Biolegend*	*343504*	*FC, cell sorting*

human CD3 Antibody FITC	*Biolegend*	*317306*	*FC, cell sorting*
human CD123 Antibody FITC	*Biolegend*	*306014*	*FC, cell sorting*
human CD1a Antibody FITC	*Biolegend*	*300104*	*FC, cell sorting*
human TCR γ/δ Antibody FITC	*Biolegend*	*331208*	*FC, cell sorting*
human TCR α/β Antibody FITC	*Biolegend*	*306706*	*FC, cell sorting*
human CD11c Antibody FITC	*Biolegend*	*301604*	*FC, cell sorting*
human CD94 Antibody FITC	*Biolegend*	*305504*	*FC, cell sorting*
human CD16 Antibody FITC	*Biolegend*	*360716*	*FC, cell sorting*
human CD303 Antibody FITC	*Biolegend*	*354208*	*FC, cell sorting*
human CD19 Antibody FITC	*Biolegend*	*302206*	*FC, cell sorting*
human CD14 Antibody FITC	*Biolegend*	*325604*	*FC, cell sorting*
human TCR FcεR1 Antibody FITC	*Biolegend*	*334608*	*FC, cell sorting*
human CD117 PE-Dazzle 594	Biolegend	313226	FC, cell sorting
human CRTH2 AF647	Biolegend	350112	FC, cell sorting
human CD161 Spark Red™ 718	Biolegend	339956	FC, cell sorting
human CD127 (IL-7Rα) PE/Cyanine7	Biolegend	351320	FC, cell sorting
human HVEM PE	Biolegend	318805	FC
human CD56 APC	eBioscience	17056742	FC, cell sorting
human CD3 FITC	eBioscience	11003742	FC
human PDL-1 PE	Biolegend	329705	FC
human HLA-E APC	Biolegend	342605	FC
human VISTA PE	Bio-Techne	FAB71261P	FC
human CD155 PE	Miltenyi Biotech	130-105-905	FC
human LIGHT PE-Cy7	Sony Biotechnologies	2193535	FC
human EpCAM PE	BD Biosciences	347198	FC
human IL22RA1 AF647	Bio-Techne	FAB2770R	FC
human αSMA (mouse)	eBioscience	1A4	IC
anti-mouse IgG AF488	Invitrogen	A21202	FC, IC
anti-rabbit IgG AF568	Invitrogen	A21206	FC
human p-Erk (rabbit)	Cell Signaling	9101S	FC
human p-S6 (rabbit)	Cell Signaling	#2211	FC
human TROP2 PE	Biolegend	363803	FC
human GPA33 AF488	Bio-Techne	FAB3080G	FC
human L1CAM AF647	Biolegend	371607	FC
human NOTCH3 AF488	Bio-Techne	FAB1559G	FC
human CD44 (mouse)	BD Biosciences	555476	FC
human Ki67 (mouse)	eBioscience	14-5699-82	FC
human IL-1R PE	Bio-Techne	FAB269P	FC
human CD142 PE	BioLegend	365204	FC

Table S2. Antibodies used in our study. FC – flow cytometry, IC – immunocytochemistry. Antibodies were used at 1:100 dilution for FC, and 1:200 for IC unless otherwise stated. Antibodies marked in *italics* are components of the lineage cocktail

**Gene**	**Primer 1**	**Primer 2**
IL17A	TAGCAGGCACAAACTCATCCA	CGTGGGATTGTGATTCCTGC
IL22	GTGCAAAAGCTGAAGGACACA	TTCTAGCAGGGAAAGGGGGT
TGFB1	CAAGCAGAGTACACACAGCA	GTATCGCCAGGAATTGTTGCT
IL6	ATTCCAAAGATGTAGCCGCC	AGTGCCTCTTTGCTGCTTTC
IL1B	AAGTACCTGAGCTCGCCAGT	CCTTGCTGTAGTGGTGGTCG

IL11	GACAAATTCCCAGCTGACGG	CGCAGGTAGGACAGTAGGT
HGF	CAAGCAATCCAGAGGTACGC	TGGTGTCTGATGATCCCAGC
EGF	CAGATCTCGATGGTGTGGGA	CCTCCATCATAATCACAGGAGC
COL1A1	AACGATGGTGCTAAGGGTGA	CCATCTTTGCCAGGAGAGCC
IL22RA1	AGAGTTCCTTGGCACCATCA	AGAGTACTGCGACGAGGAAG
IL10RB	AAAGCTGGGGAATGGAGTGA	TCTTGTAAACGCACCACAGC
IL22RA2	AGACTGTTGGGGTACTCAAGA	ACCAACAAAGAGCCATTGACT
AXIN2	CTGGCTATGTCTTTGCACCA	CTTCACACTGCGATGCATTT
LGR5	AGTGCTGTGCATTTGGAGTG	AGGGCTTTCAGGTCTTCCTC
MYC	TCCTCGGATTCTCTGCTCTC	CTCTGACCTTTTGCCAGGAG
SNAI	GATGCACATCCGAAGCCACA	TGACATCTGAGTGGGTCTGG
ZEB1	GCTGACTGTGAAGGTGTACC	ACATCCTGCTTCATCTGCCT
IFNGR1	TGACGAGATTCAGTGCCAGT	GCAACAACTGGAATCCAAAG
IFNGR2	TGCTACGAAACAATGGCAGA	AATGGGATGCTTGGTGGAGT
GAPDH	GGGTGTGAACCATGAGAAGT	CAGTGATGGCATGGACTGTG

Table S3. Primers used in our study.

Supplementary references

1. Kelemen A, Carmi I, Oszvald A et al. IFITM1 expression determines extracellular vesicle uptake in colorectal cancer. *Cell Mol Life Sci.* 2021; 78(21-22): 7009-7024.
2. Szvicsek Z, Oszvald A, Szabo L et al. Extracellular vesicle release from intestinal organoids is modulated by Apc mutation and other colorectal cancer progression factors. *Cell Mol Life Sci.* 2019; 76(12): 2463-2476.
3. Soos AA, Kelemen A, Orosz A et al. High CD142 Level Marks Tumor-Promoting Fibroblasts with Targeting Potential in Colorectal Cancer. *Int J Mol Sci.* 2023; 24(14).

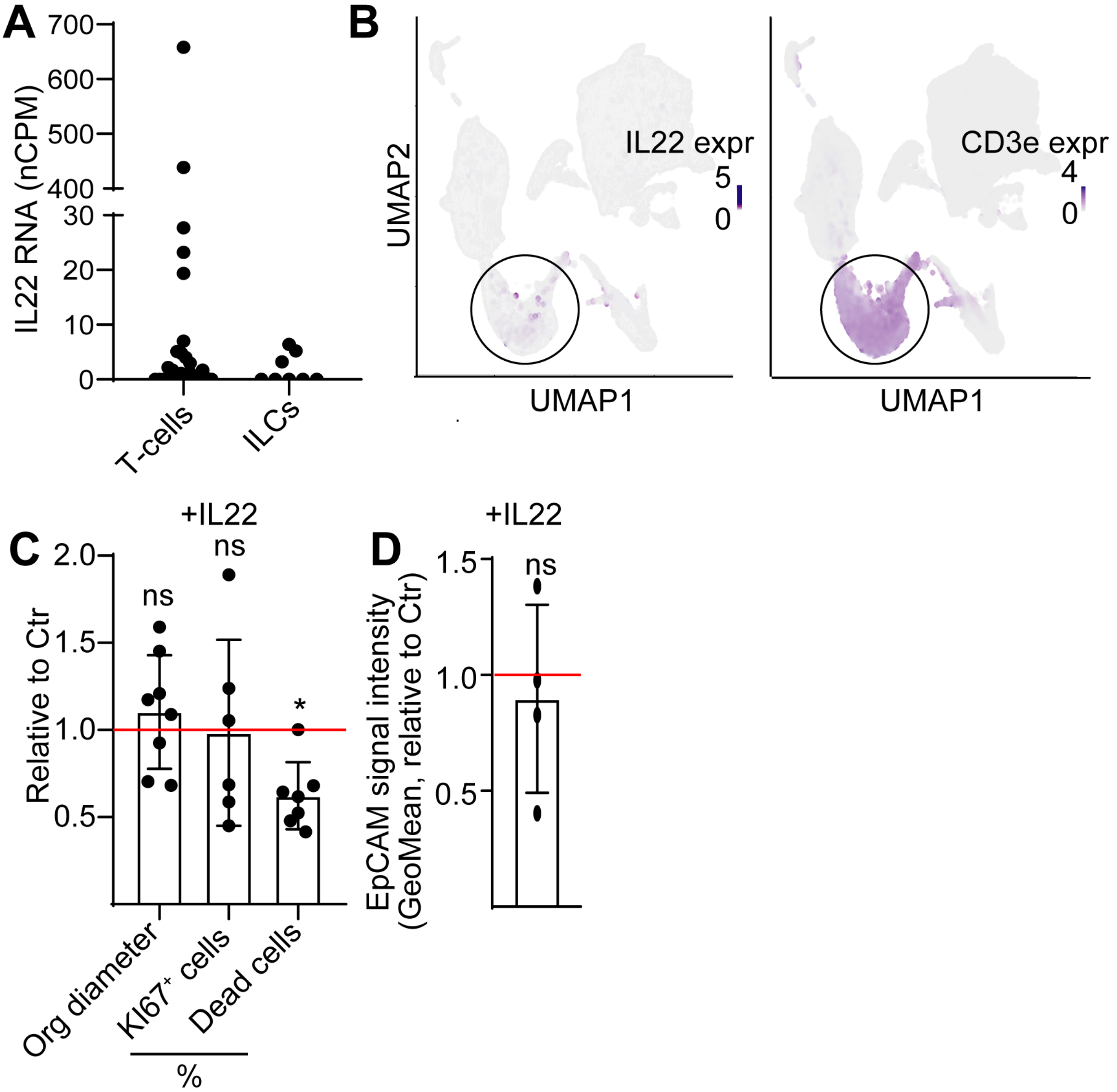

Figure S1. IL-22 is secreted by immune cells within the tumor microenvironment and does not affect cell viability and proliferation is established PDOs. A. Normalized single-cell transcriptomic data of IL-22 from the Human Protein Atlas (proteinatlas.org). Each dot represents data for T-cells or ILCs from different tissue sources. B. IL-22 and CD3e expression in CRC tissue-derived single cell RNA-seq data. The marked cluster contains T-cells (analysis by the Icarus software). Note that CD3e is a T cell marker. C. Analyis of the indicated parameters in established PDOs after treatment with IL-22 (day 3) compared to untreated controls (red line). Note that organoids were cultured 4 days before the treatment, and IL-22 was applied in the absence of EGF. Diameter was measured in light microscopy images, the ratio of KI67^{+} and dead cells was determined with flow cytometry. D. Signal intensity of cell surface EpCAM level in IL-22-treated organoids (added on day 4 for 3 days) compared to untreated controls (flow cytometry). Each dot represents one PDO line (C-D). In C, PDO lines were measured multiple times. Paired t-test with *$p<0.05$, ns: $p>0.05$

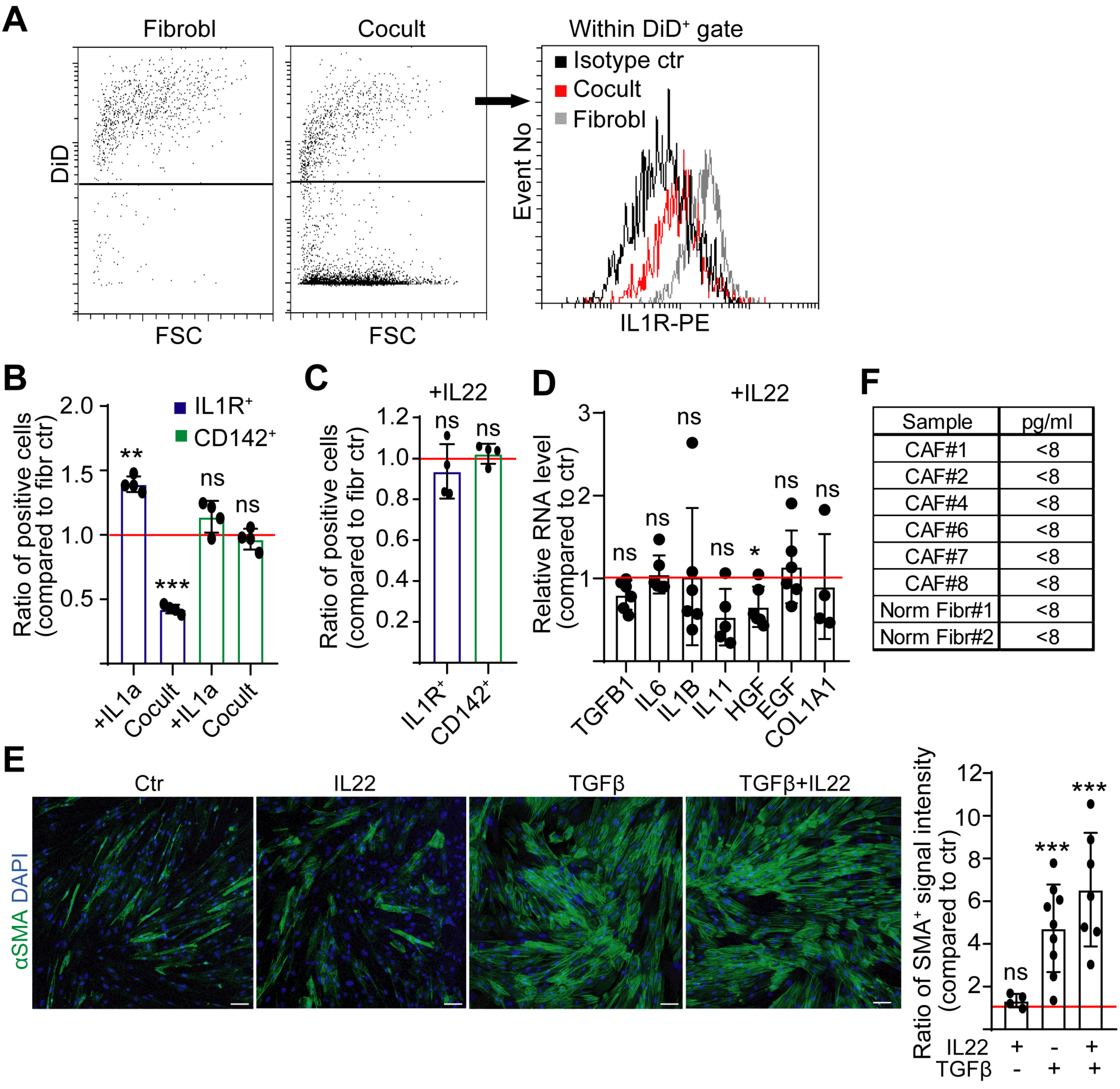

Figure S2. Fibroblast polarization remains unaffected in the presence of IL-22. A. Flow cytometry showing IL1R$^+$ fibroblasts marked with the fluorescent DiD membrane-labelling dye in 3D cultures with only fibroblasts and their co-cultures with PDOs. Note the lower percentage of IL1R$^+$ cells within the DiD$^+$ fibroblast gate in co-cultures (right panel). B. The relative ratio of IL-1R$^+$ and CD142$^+$ cells within the fibroblast gate in cultures treated with IL-1α or in co-cultures with PDOs, compared to fibroblast monocultures as a control (red line). Note the effect of the positive control IL-1α on the IL1R$^+$, but not the CD142$^+$ population. C. The relative ratio of IL-1R$^+$ and CD142$^+$ cells within the fibroblast gate in the presence of IL-22 (50 ng/mL for 3 days, flow cytometry). D. The relative RNA levels of the indicated markers in fibroblasts treated with IL-22 (RT-qPCR). RNA levels were normalized to housekeeping and then compared to the untreated controls (red line). E. Confocal microscopy images of fibroblasts with the indicated treatments for αSMA (left panels), and their quantification (right panel). F . IL-22 levels in CAF and normal fibroblast (ATCC-1459 cell line) conditioned media (ELISA). Each dot represents one PDO (B, C) or one measurement (D, E). For D, PDO lines were measured 1-2 times. Paired (A-D) or unpaired (E) t-test with ***$p<0.005$, **$p<0.01$, *$p<0.05$, ns: $p>0.05$

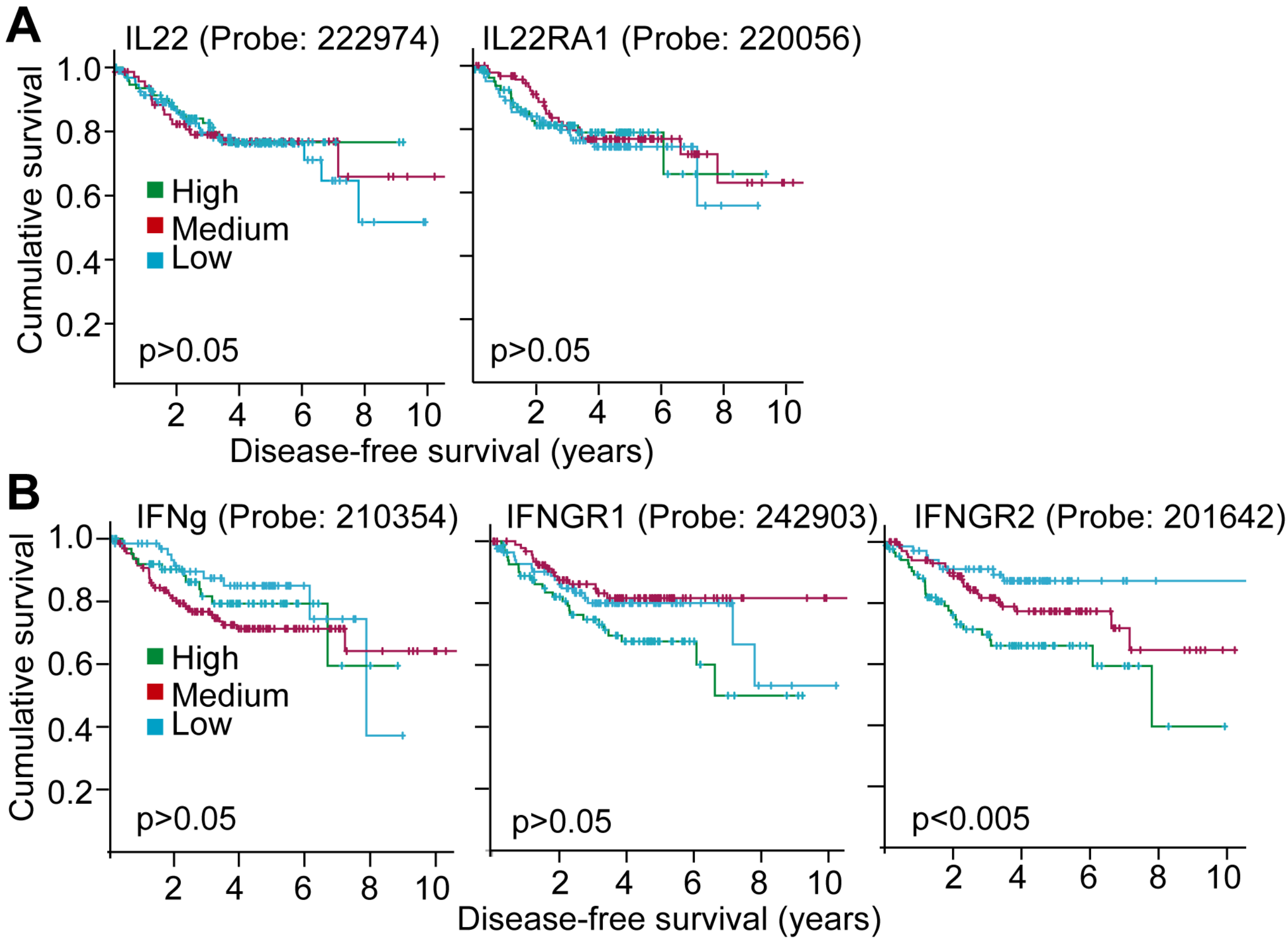

Figure S3. IL22, IL22RA1, IFNγ and IFNGR1 do not show correlation with patient survival. A-B. Kaplan-Meier survival curves for the indicated genes and probes with log-rank test p-values (see Materials for details of the analysed data sets)
